# Efficacy of Surgical Reconstruction With Rectus Abdominis Flap Following en Bloc Resection of the Orbital Contents for Malignant Lacrimal Gland Cancer: A Case Series

**DOI:** 10.7759/cureus.69310

**Published:** 2024-09-13

**Authors:** Misato Ueda, Hirotaka Shinomiya, Ken-ichi Nibu, Hidehito Kimura, Tadashi Nomura

**Affiliations:** 1 Department of Plastic Surgery, Kobe University Graduate School of Medicine, Kobe, JPN; 2 Department of Otolaryngology - Head and Neck Surgery, Kobe University Graduate School of Medicine, Kobe, JPN; 3 Department of Neurosurgery, Kobe University Graduate School of Medicine, Kobe, JPN

**Keywords:** free flap transfer, lacrimal gland tumor, orbital exenteration, reconstruction of skull base, rectus abdominis flap

## Abstract

In cases of lacrimal gland carcinoma requiring surgical excision of the orbital contents, skull base, and surrounding bones, definitive blockage of the cranial cavity and reconstruction of the anterior skull base with irradiation-acceptable tissue (for possible subsequent radiotherapy) is necessary. However, considerations for quality of life, including cosmetic aspects, such as artificial eye placement and contour morphology, make reconstruction challenging. In three cases of advanced lacrimal gland carcinoma, we performed a reconstruction surgery following an en bloc resection of the orbital contents and lateral orbital bones. A rectus abdominis flap was used, considering both function and morphology. This flap, characterized by reliable anatomical structure and good blood flow, adequately filled the three-dimensional dead space. In our case, the flap fully survived, and no complications such as cerebrospinal fluid leakage or meningitis were observed. Six months after surgery, the flap volume was 31.7-73.3% of its initial size. Considering potential flap shrinkage in the future, it was deemed beneficial to use a slightly excessive volume.

## Introduction

Lacrimal gland carcinoma is a rare malignancy [1]. Depending on the stage of the disease, en bloc surgical resection including that of orbital contents, the skull base, and surrounding bones, may be required [1]. In such cases, a reliable blockade of the cranial cavity and subsequent reconstructive measures for the anterior skull base with tissues acceptable for radiotherapy may be necessary. Consideration of aesthetic aspects, such as prosthetic eye socket formation and contour morphology, are also essential to ensure quality of life; however, this makes reconstruction challenging [2]. Herein, we report our experience with three patients with lacrimal gland carcinomas who underwent en bloc resection, including that of orbital contents and bones of the lateral orbital wall, followed by anterior skull base reconstruction. We discuss the positioning of and volume changes in the skin flaps.

This article was previously presented as a meeting presentation at the 47th Annual Meeting of the Japanese Society for Head and Neck Cancer on June 15-16, 2023.

## Case presentation

**Figure 1:**
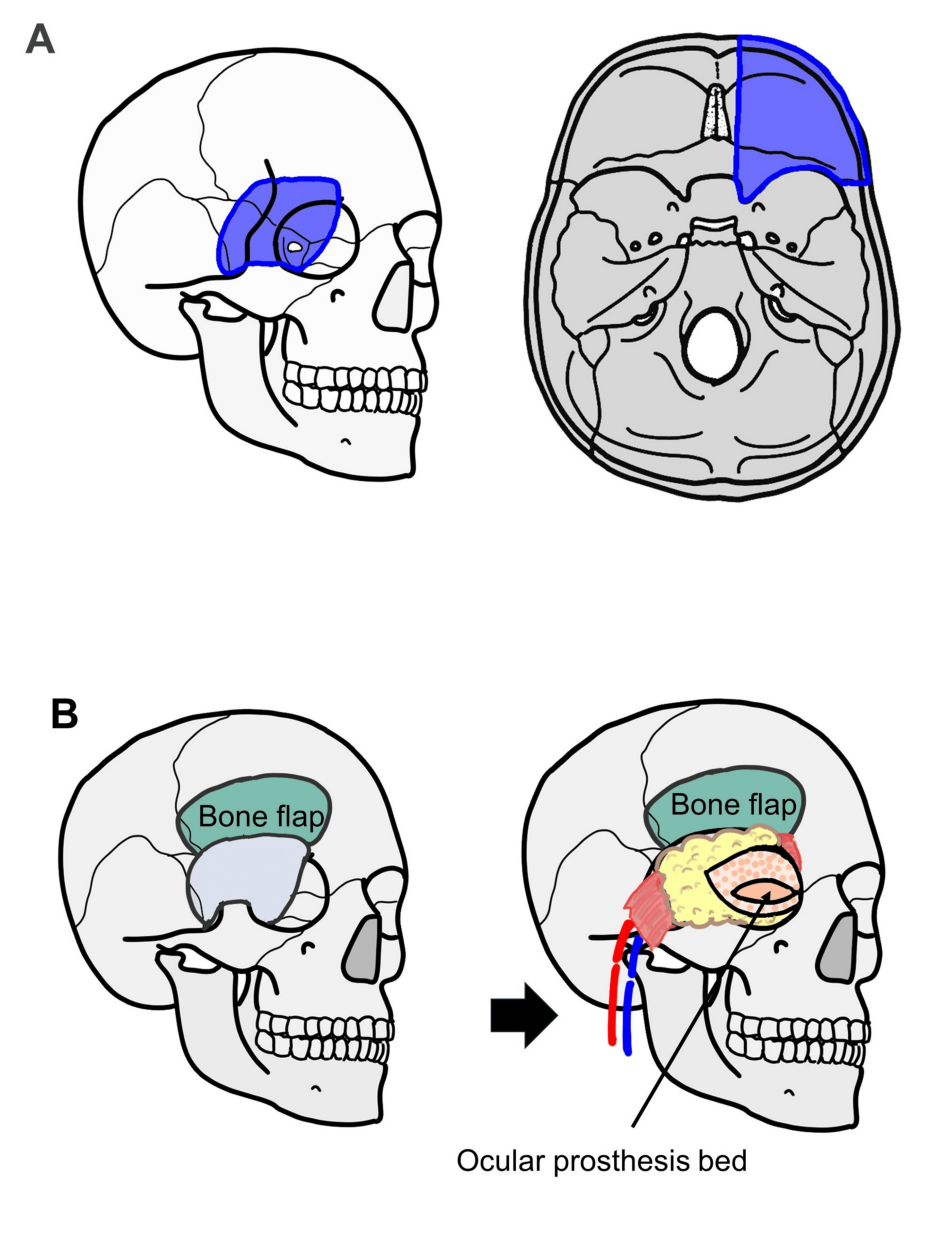
Surgical schema and clinical findings of reconstruction with rectus abdominis flap (A) The extent of resection includes the upper lateral wall of the orbit and the frontal skull base (blue area). The anterior clinoid process is drilled, and the ophthalmic branch of the trigeminal nerve, optic nerve, ophthalmic artery, oculomotor nerve, trochlear nerve, abducens nerve, and supraorbital nerve are severed. (B) The autologous bone flap removed during anterior skull base surgery was replaced. The dural defect was reconstructed using a pericranial flap. The superficial temporal and deep inferior epigastric vessels are anastomosed, and a free rectus abdominis flap is transplanted into the defect area. A socket is created for the prosthetic eye. Image Credits: Misato Ueda.

Patient consent has been obtained for publication of the medical images included in this report. This report further adheres to the ethical principles outlined in the Declaration of Helsinki (as amended in 2013). All three patients were men, with ages ranging from 32 years to 70 years (mean age, 46 years) at the time of surgery. Pathologically, one patient was diagnosed with adenocarcinoma, whereas two patients were diagnosed with adenoid cystic carcinoma. Two patients underwent postoperative radiotherapy, and one patient had a history of preoperative radiotherapy. Reconstruction was performed using a free rectus abdominis flap in all patients, with no rigid reconstruction using autogenous or artificial bone (Table 1). A multidisciplinary team comprising otolaryngologists, neurosurgeons, and plastic surgeons was involved in the surgery. An en bloc resection of the orbital contents and the lateral orbital wall and frontal skull base surgery was performed in all cases (Figure 1A). The dural defect was reconstructed using a pericranial flap at the site where the defect occurred. Reconstructive surgery was performed using a free rectus abdominis flap, positioned to ensure a reliable separation between the anterior cranial fossa and the reconstructed area. In addition, the flap was embedded subcutaneously and used to fill the dead space in the defect area and shaped with a slightly excess volume to account for postoperative shrinkage. The skin of the flap was exposed in the eye area to create a socket for the prosthetic eye. No rigid reconstruction using autogenous or artificial bone was performed (Figure 1B). In one patient, venous thrombosis was observed at the anastomotic site, requiring re-anastomosis. However, the flaps in all patients survived successfully. No patient experienced any complications, such as cerebrospinal fluid leakage or meningitis postoperatively.

**Table 1:** Patient and treatment characteristics

Case	Age (y)	Sex	Pathological diagnosis	Postoperative radiotherapy	Postoperative chemotherapy	Reconstructive procedure
1	70	M	Lacrimal gland adenoid cystic carcinoma recurrence	(70.4 Gy before recurrence)	－	Free rectus abdominis flap
2	32	M	Poorly differentiated adenocarcinoma	66 Gy	＋	Free rectus abdominis flap
3	36	M	Lacrimal gland adenoid cystic carcinoma	70.4 Gy	―	Free rectus abdominis flap

**Figure 2:**
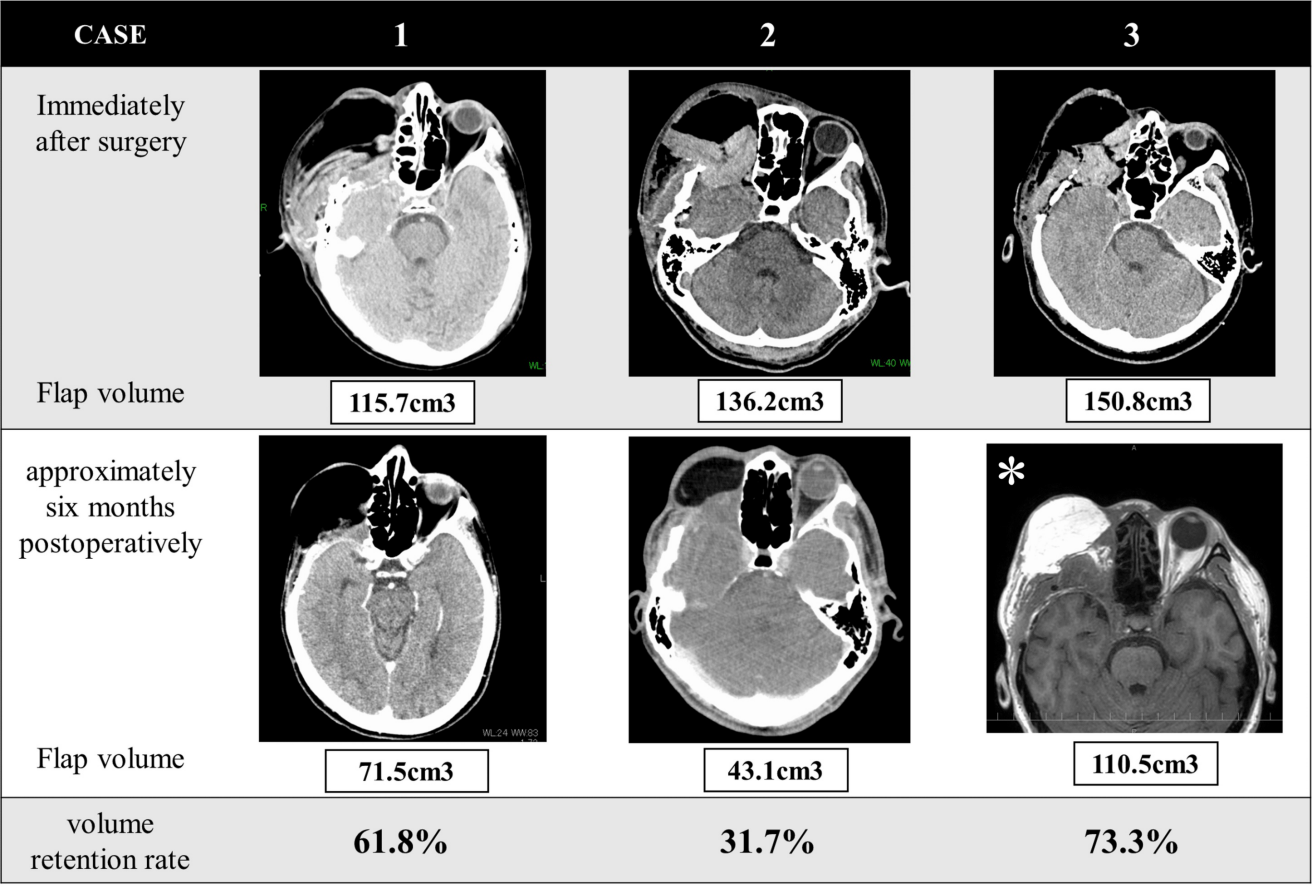
Volume change of the free flap The volumes of the transplanted rectus abdominis flaps are observed immediately after surgery and approximately 6 months postoperatively. The volume retention rates at approximately 6 months postoperatively were 61.8% for case 1, 31.7% for case 2, and 73.3% for case 3. Flap volumes were evaluated through image processing using the OsiriX Dicom Viewer (Pixmeo Inc., Genève, Switzerland). *Since CT imaging was not performed during the relevant period, the volume of the flap was measured using MRI images.

Flap volumes were evaluated through image processing using the OsiriX Dicom Viewer (Pixmeo Inc., Genève, Switzerland). The details of the cases are presented in Figure 2.

Case 1

**Figure 3:**
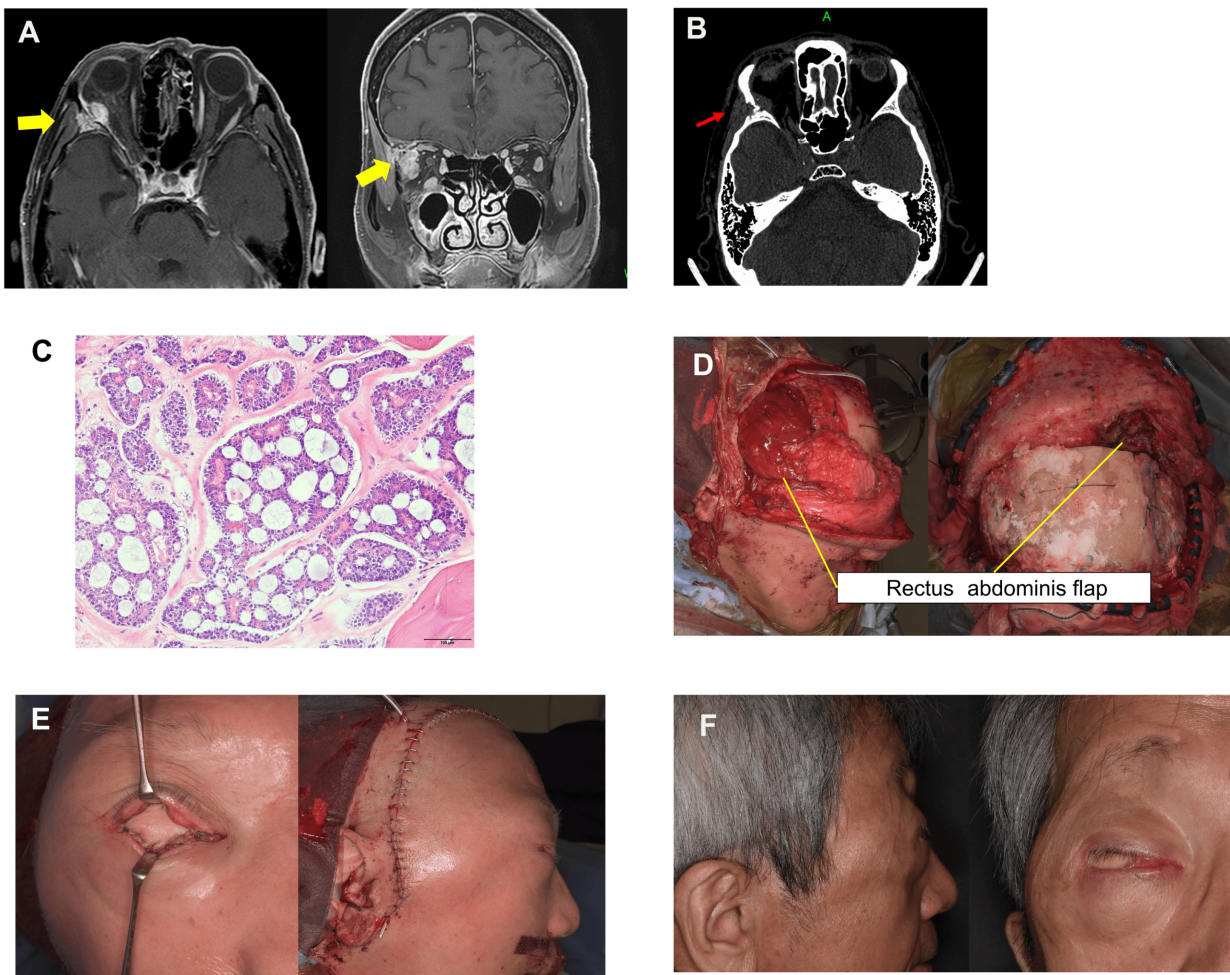
Case 1 (A) On contrast-enhanced T1-weighted magnetic resonance imaging (MRI), a mass with contrast enhancement was observed on the lateral side of the right orbit. Contrast enhancement is also noted in the lateral rectus muscle (yellow arrow). (B) Bone destruction is observed around the tumor in the orbit (red arrow). (C) Hematoxylin and eosin (HE) staining reveals the formation of cell nests accompanied by small cysts. This finding is consistent with the cribriform pattern of adenoid cystic carcinoma. (×100). (D) The superficial temporal and deep inferior epigastric vessels are anastomosed, and a free rectus abdominis flap is transplanted into the defect area. A socket is created for the prosthetic eye. (E) Clinical findings immediately after the reconstruction surgery. (F) Clinical appearance 9 months after reconstruction. The flap volume has decreased, whereas the volume in the eye area remains excessive.

The patient was a 70-year-old male with a history of proton beam therapy for right lacrimal gland adenoid cystic carcinoma two and a half years prior to the surgery. During follow-up, contrast-enhanced T1-weighted MRI revealed a mass with contrast enhancement on the lateral side of the right orbit along with enhancement in the lateral rectus muscle (Figure 3A). CT showed bone destruction around the tumor in the orbit (Figure 3B). A partial biopsy confirmed the diagnosis of adenoid cystic carcinoma (Figure 3C). A multidisciplinary team, including otolaryngologists, neurosurgeons, and plastic surgeons, was involved in the surgery. An en bloc resection of the orbital contents and the lateral orbital wall and frontal skull base surgery was performed. A rectus abdominis flap was harvested from the right abdomen. The recipient vessels were the superficial temporal artery and anastomosed with the deep inferior epigastric vessels. The flap was positioned to ensure a reliable blockade between the anterior cranial fossa and the reconstructed area. Moreover, the flap was embedded subcutaneously and used to fill the dead space in the defect area, with a slightly excess volume to account for postoperative shrinkage. The skin of the flap, except for the ocular prosthesis bed, was de-epithelialized before the flap was placed. A part of the flap skin paddle was exposed in the eye area to create a socket for the prosthetic eye. (Figures 3D-3E). The flap completely survived. Postoperatively, no complications, such as cerebrospinal fluid leakage or meningitis, were observed. Over time, the flap volume decreased (Figure 3F). Although the volume in the eye area remained prominent, the patient declined to undergo revision surgery.

Case 2

**Figure 4:**
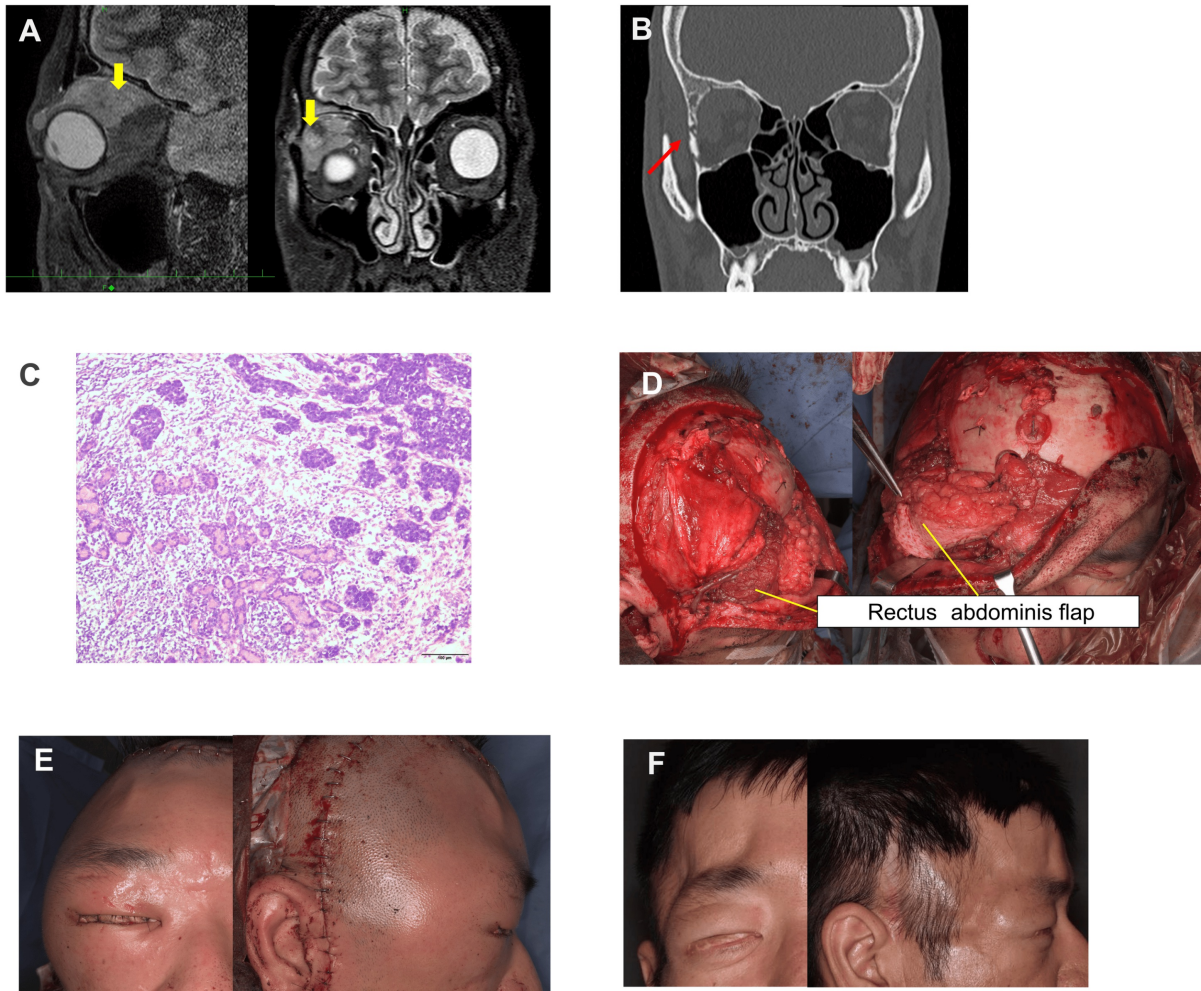
Case 2 (A) A 23-mm high-signal tumor involving the adjacent extraocular muscle is observed on short-tau inversion recovery (STIR) imaging of the lacrimal gland (yellow arrow). (B) Bone destruction around the tumor in the orbit is observed (red arrow). (C) Hematoxylin and eosin staining shows atypical cells with a high nuclear-to-cytoplasmic (N/C) ratio that irregularly formed small nests, and small glandular structures are observed in some areas. The diagnosis was poorly differentiated adenocarcinoma (×100). (D) The superficial temporal and deep inferior epigastric vessels are anastomosed, and a free rectus abdominis flap is transplanted into the defect area. A socket is created for the prosthetic eye. (E) Clinical findings immediately after the reconstruction surgery. (F) Clinical appearance 7 months after reconstruction. The volume of the transplanted flap has decreased, with mild concavity observed in the forehead and temple areas.

A 32-year-old man presented to our hospital with swelling and limited movement of the right eye. MRI revealed a 23 mm lesion with high-signal intensity involving the lacrimal gland and adjacent extraocular muscles on short-tau inversion recovery (STIR) images (Figure 4A). CT scan revealed bone destruction in the orbit around the tumor (Figure 4B). Histopathologically, the patient was diagnosed with a poorly differentiated lacrimal gland carcinoma based on a partial biopsy (Figure 4C). A multidisciplinary team comprising otolaryngologists, neurosurgeons, and plastic surgeons was involved in the surgical procedures. An en bloc resection of the orbital contents and lateral wall of the orbit (Figure 4D), followed by anterior skull base reconstruction, was performed. Subsequently, a reconstructive surgery was performed. The rectus abdominis flap was harvested from the right abdominal region. The recipient vessels were the superficial temporal artery and anastomosed with the deep inferior epigastric vessels. The flap was positioned to ensure reliable blockade between the anterior cranial fossa and the reconstructed area. Additionally, the flap was denuded, and the dead space in the defect area was filled with the flap; the flap was trimmed and shaped to fit the defect with a slightly excess volume to account for postoperative shrinkage. A skin island was exposed in the eye area to create a socket for the prosthetic eye (Figures 4D-4E). The day after surgery, venous thrombosis was observed at the anastomotic site, requiring re-anastomosis. There were no clear findings of compression on the vascular pedicle by the flap. In a salvage procedure, a graft from the external jugular vein to the end of the flap was used. However, the flap survived in its entirety. Postoperatively, no complications (such as cerebrospinal fluid leakage or meningitis) were observed. The patient underwent postoperative radiotherapy and chemotherapy. Over time, the flap volume reduced to an appropriate size (Figure 4F). Mild concavity was noted in the temple and forehead areas, and future touch-up procedures, such as fat injections, were planned. Furthermore, adjustments to the prosthetic eye were scheduled.

Case 3

**Figure 5:**
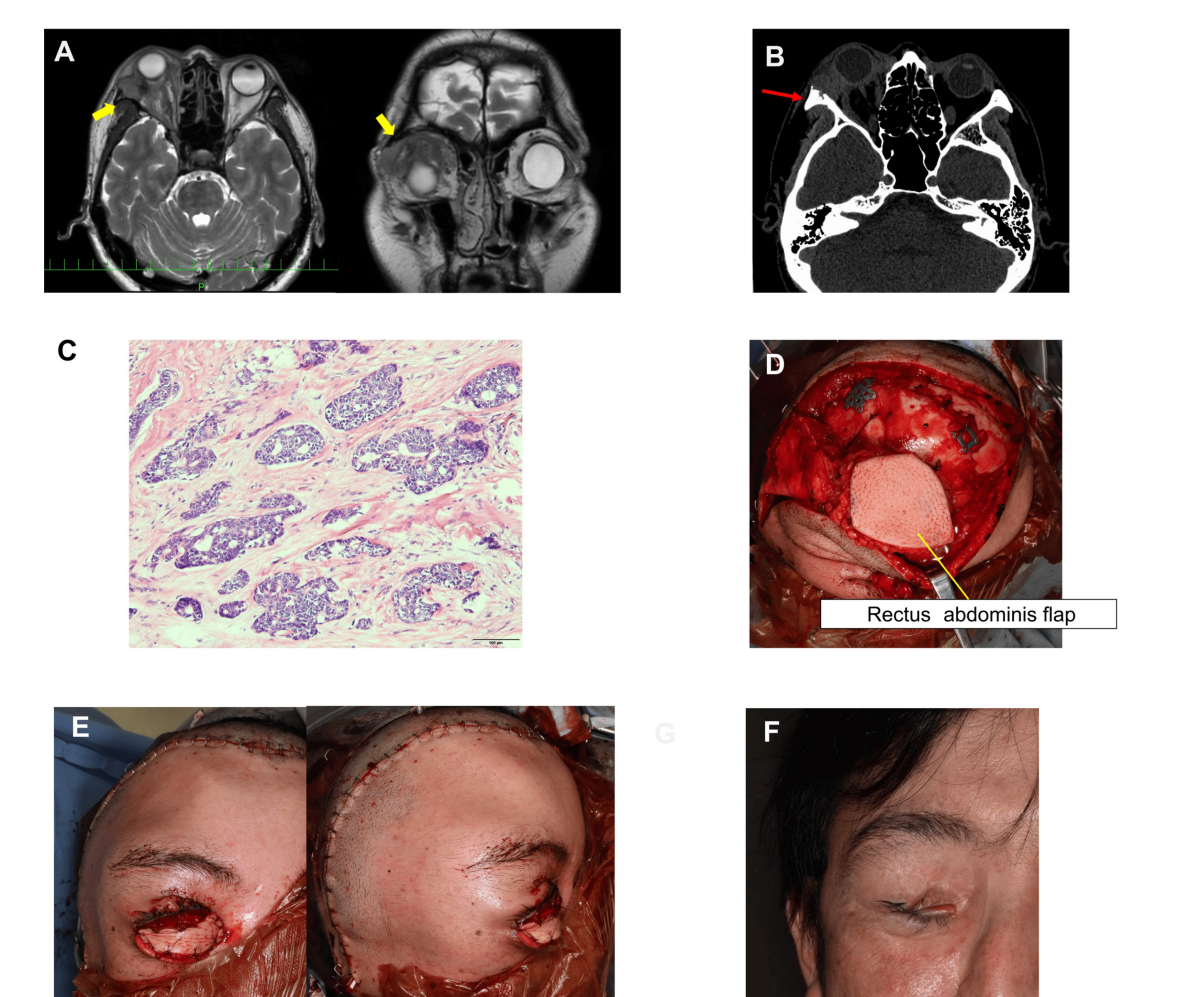
Case 3 (A) On T2-weighted magnetic resonance imaging (MRI), a mass lesion with irregular margins and unclear boundaries, ranging from mildly hyperintense to hypointense, is observed in the right orbit, lacrimal gland, and eyelid. (yellow arrow). (B) Bone destruction is observed around the tumor in the orbit (red arrow). (C) Hematoxylin and eosin staining reveals infiltrative proliferation of atypical epithelium with a trabecular and anastomosing pattern, displaying a biphasic tubular structure, accompanied by marked sclerosis in the background (×100). (D) The superficial temporal and deep inferior epigastric vessels are anastomosed, and a free rectus abdominis flap is transplanted into the defect area. A socket is created for the prosthetic eye. (E) Clinical findings immediately after the reconstruction surgery. (F) Clinical appearance 1 year and 5 months after reconstruction. The patient underwent two revision surgeries.

A 36-year-old male presented to a local clinic with swelling and difficulty closing his right eye, prompting a referral to our hospital for a suspected orbital tumor. MRI revealed a mass lesion with irregular margins and unclear boundaries in the right orbit, lacrimal gland, and eyelid. The borders between the tumor and the extraocular muscles, as well as the optic nerve, were indistinct, raising suspicion of invasion (Figure 5A). CT scan showed bone destruction on the lateral side of the right orbit (Figure 5B). A partial biopsy confirmed the diagnosis of adenoid cystic carcinoma of the lacrimal gland (Figure 5C). A multidisciplinary team, including otolaryngologists, neurosurgeons, and plastic surgeons, was involved in the surgery. An en bloc resection of the orbital contents and the lateral orbital wall and frontal skull base surgery was performed. Subsequently, frontal skull base reconstruction was performed. A rectus abdominis flap was harvested from the right abdomen. The recipient vessels were the right superficial temporal artery, and the flap was anastomosed to the deep inferior epigastric artery. The flap was positioned to ensure a reliable blockade between the anterior cranial fossa and the reconstructed area. Moreover, the flap was embedded subcutaneously and used to fill the dead space in the defect area, with a slightly excess volume to account for postoperative shrinkage. A skin island was exposed in the eye area to create a socket for the prosthetic eye (Figures 5D-5E). The upper eyelid was partially reconstructed due to its missing portion. The flap completely survived, and no postoperative complications, such as cerebrospinal fluid leakage or meningitis, were observed. The patient underwent postoperative radiotherapy. Over time, the flap volume decreased to an appropriate size. Two revision surgeries were performed to adjust the contour of the eye area (Figure 5F). However, plans for a prosthetic eye socket formation were postponed owing to metastasis to the cervical spine.

## Discussion

Radical orbital exenteration for locally advanced lacrimal gland carcinoma often results in distinct three-dimensional structural defects. This procedure involves resection of the anterior skull base and frontal-temporal bone, including the orbital contents and upper lateral orbital bones. In such cases, reconstruction of the anterior skull base is essential but presents a challenge [1]. The defects resulting from such radical surgeries have complex morphologies, extending from the eye socket to the temporal and frontal regions. Achieving a reliable blockade between the cranial cavity and filling the dead spaces requires a certain volume of skin flap. Additionally, the reconstruction must withstand radiation therapy and provide vascularized tissue suitable for cranial base reconstruction [2]. Considering the impact on facial contours due to the removal of the upper and lateral orbital walls and the absence of the eyeball, prosthetic eye socket reconstruction is necessary for subsequent artificial eye insertion. The goal is to achieve reconstruction that addresses not only functional aspects but also aesthetic concerns. Reports on free flap reconstruction for defects in the skull base and orbital bones include the use of the rectus abdominis, anterolateral thigh, forearm, and latissimus dorsi flaps [2]. Herein, we opted for the rectus abdominis flap because of its stable blood flow, ability to use a long vascular pedicle, and flexibility in achieving various shapes and volumes. Some reports have suggested that extensive soft tissue transfer with a rectus abdominis flap eliminates the need for bone reconstruction [3]. Although we did not perform bone reconstruction in this case, the patient may have experienced concavity in the bone defect area over time. Accordingly, we planned additional interventions, such as fat injections, based on patient preferences. The flap volume changes over time; for free rectus abdominis flaps, the residual flap volume ranges from 55.8% to 76.9% 1 year postoperatively [4-6]. Factors contributing to flap volume reduction include denervation, ischemia, and body weight reduction [7-10]. The reduction in flap volume is largely influenced by muscle tissue, and flaps with a higher fat-to-muscle ratio tend to be better preserved [4-6]. There are conflicting reports regarding the effects of postoperative radiation therapy on flaps. Some studies have suggested that radiation therapy does not affect the volumes of deep inferior epigastric perforator flaps in breast reconstruction [11]. Yamaguchi et al. [4] stated that radiation therapy did not significantly affect the volume of adipose tissue in flaps, which was attributed to the condition of the host. Kimura et al. [9] reported that radiation therapy did not affect the total flap volume. Conversely, Sakakibara et al. [5] reported a significant decrease in the total flap volume due to radiation therapy. Similarly, Sakamoto et al. [6] suggested that radiation is a significant factor affecting flap volume. Particularly, the volume reduction in the irradiated group was markedly increased compared with that in the non-irradiated group, especially in the muscle tissues. We chose the rectus abdominis flap because of the ease of tissue filling and the potential for shrinkage over time. Sufficient tissue was available to fill the defect, and no dead space was present within the cranial cavity; this allowed for a reliable blockade between the cranial cavity and the reconstructed area. Because the orbital floor remained intact, the volume of the rectus abdominis flap was less likely to cause ptosis. We planned to reduce the orbital volume to achieve the desired morphology. In our department, we have adopted a two-stage approach for bony reconstruction procedures such as free bone grafting; these procedures are not performed immediately but are rather deferred to a later stage to facilitate prompt transition to adjuvant therapy. Owing to the absence of bone, there was a mild concavity in the contour of the lateral wall of the orbit. Depending on the need, procedures such as fat injection or modifications to the eyelids and prosthetic eye sockets may be considered. Although the anterolateral thigh (ALT) flap is rich in adipose tissue, leading to less volume reduction, it is a perforator flap. In our three cases, we opted for reconstruction using the rectus abdominis flap, which is considered to have a more stable blood flow. During reconstruction, we intentionally used a slightly excessive flap volume, which became relatively suitable over time. When positioning the flap, we generally avoid placing it on the convexity dura to prevent brain compression and instead place it on the cranial base side. We further ensured that it is confined to the orbital region. However, considering the potential for further shrinkage of the flap, we will continue to monitor its impact on the contour, and further assessments of flap volume changes will be conducted in the future. Reconstructive surgery for advanced lacrimal gland carcinoma involving en bloc resection of the orbital contents and lateral wall of the orbit was performed considering both function and morphology. The reconstruction used a free rectus abdominis flap, with a deliberate slight excess flap volume to account for subsequent shrinkage.

## Conclusions

In patients with advanced lacrimal gland carcinomas, reconstructive surgery involving an en bloc resection of the orbital contents and lateral wall should be performed considering both function and morphology. Use of a free rectus abdominis flap with a slightly excess flap volume, deliberately retained to account for subsequent shrinkage, may achieve a secure barrier with the cranial cavity, fill dead spaces, and allow for aesthetic considerations.
